# Supplementary material for: A set of multi-entry identification keys to African frugivorous flies (Diptera, Tephritidae)
Source: Zookeys. 2014 Jul 24;(428):97–108. doi: 10.3897/zookeys.428.7366 (PMC4143993; doi:10.3897/zookeys.428.7366)
Supplement: Supplementary material 6 — Key to Ceratitis [file zookeys-428-097-s006.zip › SF6_ZooKeys_key to Ceratitis/key/SF6_ZooKeys_key to Ceratitis/Media/Html/Ceratitis roubaudi .htm]

Ceratitis (Ceratalaspis) roubaudi (Bezzi)


***Ceratitis*** **(*Pterandrus*) *roubaudi*
(Bezzi)**

*Pardalaspis roubaudi* Bezzi, 1923: 527.

Body
length: 5.13 (4.40-6.15) mm; wing length: 5.04 (4.60-5.40) mm.

Male

Head: Antenna orange to brown (yellow according to the
original description). First flagellomere twice as long as pedicel. Arista with
short rays (medium long rays according to the original description); ventral
rays shorter and sparser than dorsal rays, especially basally. Frons convex;
dark yellow, with short scattered setulae distinctly darker than frons. Face
white; gena darker yellow. Genal seta and setulae dark, well developed.

Thorax:
Postpronotal lobe white (yellow according to the original description), with
brown spot. Scutal pattern, ground color dark brown, microtrichose areas
silvery with ashgray shine; prescutellar white markings separate, with pale
gray area in between. Scapular setae dark. Scutellum white, basally with two
separate dark spots (not mentioned in original description), apically with
three separate black spots, reaching to basal 0.3 of scutellum. Anepisternum
yellow, ventral half brown; one anepisternal seta; anepisternal setulae pale,
except in ventral half.

Legs:
Yellowish brownish (yellow  according to original description); setation
typical for subgenus, mainly black; no feathering.

Wing:
Bands brown. Marginal band forming continuous band with anterior part of discal
band; cubital band free; medial band absent; crossvein R-M just proximal of 
middle of discal cell. Apex of vein R_1_ distal to level of crossvein
R-M. Crossvein DM-Cu oblique anterobasally.

Abdomen:
Mostly pale orange. Tergites 2 and 4 with gray transverse band along  posterior
half, brownish anteriorly. Tergite 3 largely brown. Setation typical for
subgenus. Male terminalia not dissected.

Female

As
male except for the following characters: One or two anepisternal setae
present. Crossvein R-M opposite middle of discal cell. Oviscape shorter than
preabdomen. Aculeus about six times longer than wide; tip pointed and lateral
margin slightly concave.

(Description
after De Meyer & Freidberg, 2006)
